# Infection-provoked reversible posterior leukoencephalopathy syndrome in an adult with nephrotic syndrome: a case report

**DOI:** 10.1186/s12883-020-01922-x

**Published:** 2020-09-17

**Authors:** Kuan-Ying Li, Ching-Fang Chien, Chin-Ling Tsai, Huang-Chi Chen, Meng-Ni Wu, Chiou-Lian Lai, Li-Min Liou

**Affiliations:** 1 Department of Neurology, Kaohsiung Medical University Hospital, Kaohsiung Medical University, Kaohsiung, Taiwan; 2 Department of Neurology, School of Medicine, College of Medicine, Kaohsiung Medical University, Kaohsiung, Taiwan; 3 Department of Neurology, Kaohsiung Municipal Siaogang Hospital, Kaohsiung Medical University, Kaohsiung, Taiwan; 4 Department of Internal Medicine, Kaohsiung Municipal Hsiao-Kang Hospital, Kaohsiung Medical University, Kaohsiung, Taiwan; 5 Division of Pulmonary and Critical Care Medicine, Department of Internal Medicine, Kaohsiung Medical University Hospital, Kaohsiung Medical University, Kaohsiung, Taiwan

**Keywords:** Reversible posterior leukoencephalopathy syndrome, Nephrotic syndrome, Infection

## Abstract

**Background:**

Reversible posterior leukoencephalopathy syndrome (RPLS) is a rare and heterogeneous clinico-neuroradiological syndrome characterized by headache, altered mental status, seizures, and visual disturbances. Hypertension and immunosuppression are two of the main factors that predispose an individual to RPLS. However, RPLS can develop when no major risk factors are present. RPLS has been reported in pediatric nephrotic patients, but rarely in adults.

**Case presentation:**

A 42-year-old Asian woman with nephrotic syndrome presented with seizures, headaches, and nausea. Her blood pressure was controlled, and no immunosuppressants had been prescribed. All symptoms and tests indicated RPLS following infection with pneumonia, which was successfully treated by immediate administration antibiotic and anti-epileptic medications. Seizures did not recur during a 2-year follow-up period.

**Conclusions:**

When patients with nephrotic syndrome have an infection, RPLS symptoms should be investigated thoroughly. With early diagnosis and appropriate treatment of RPLS, morbidity and mortality can be prevented.

## Background

Reversible posterior leukoencephalopathy syndrome (RPLS) is a rare and heterogeneous clinico-neuroradiological syndrome characterized by the rapid onset of headaches, altered mental status, seizures, and visual disturbances associated with reversible white matter changes. The condition is diagnosed using neuroimaging, although clinicians must first suspect the condition based on the patient’s symptoms. With timely diagnosis, RPLS can be resolved within a few days to a week; hence, it must be recognized and treated promptly.

RPLS has been reported in pediatric nephrotic patients, but rarely in adults [1]. Hypertension and immunosuppression are two of the main factors that predispose an individual to RPLS. Specifically, 70% of patients experience acute hypertension immediately before RPLS onset [2], and infections have been observed [3]. In the present report, we describe an unusual case of RPLS as a result of infection in an adult with nephrotic syndrome who had normal blood pressure (BP).

## Case presentation

**Fig. 1:**
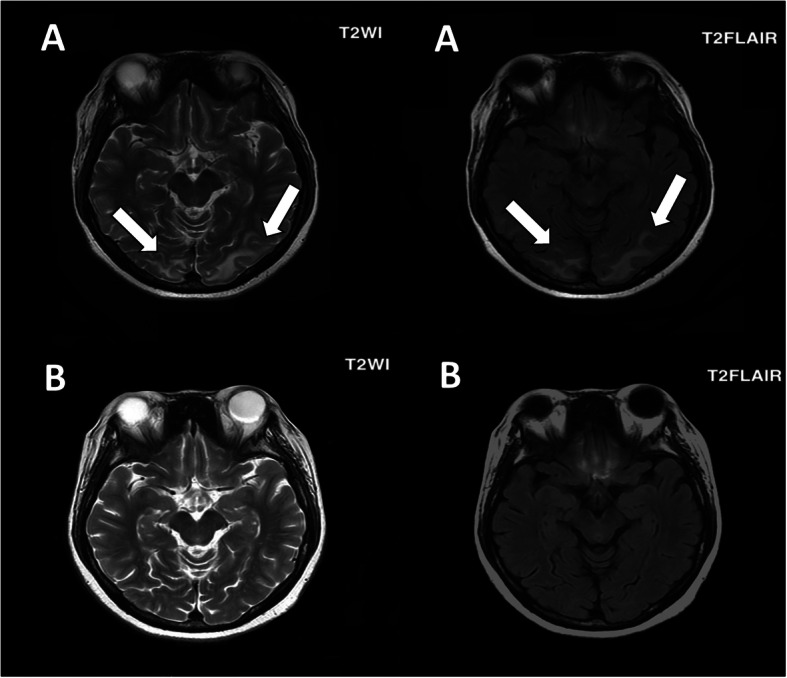
**a** High signal intensity on magnetic resonance (MR), T2-weighted imaging and MR T2-weighted fluid-attenuated inversion recovery imaging in the parietal–occipital lobes observed 2 days after the seizure episodes. **b** The lesions had disappeared 3 months later

A 42-year-old Asian woman presented to the emergency room with generalized tonic-clonic seizures, headache, nausea, and vomiting. Specifically, she had experienced four episodes of 5-min seizure without evident aura before admission, followed by 30 min of postictal confusion. This was her first presentation of seizure. A medical history of nephrotic syndrome probably caused by immunoglobulin A nephropathy and hypertension was recorded. Since the patient refused to undergo renal biopsy, the exact diagnosis of immunoglobulin A nephropathy could not be confirmed. Her family history was unremarkable, and she received only antihypertensive medications and a moderate dose of prednisolone (45 mg per day) for immunoglobulin A nephropathy before presentation. She also reported cough with mild dyspnea 1 week prior to the onset of seizures. On admission, physical examination revealed that the patient was afebrile (temperature: 36.5 °C) with a BP of 141/85 mmHg, a heart rate of 110 beats/minute, and markedly decreased breath sounds over the right lung. No neurological deficits, other than mild postictal sleepiness, were demonstrated on neurological examination. Laboratory investigations demonstrated leukocytosis with left shift (leukocyte count: 23.21 × 10^4^, neutrophil count: 86.9%) and an elevated C-reactive protein level (33 mg/dL), although blood sugar level, electrolytes, and renal and liver function were normal. Urinalysis revealed heavy proteinuria (spot urine: > 3+). Notably, the patient had severe hypoalbuminemia (1.51 g/dL), with serum albumin at its lowest level ever recorded in the patient. Chest radiography revealed abundant right-side pleural effusion superimposed with lung infiltration. To treat this condition, thoracentesis was performed several days after admission. The patient’s pleural fluid was cloudy, and studies revealed lactic acid dehydrogenase levels of 187 U/L (serum value: 271 U/L) and total protein levels of 4.1 g/dL (serum value: 1.1 g/dL), indicating exudative pleural effusion. Brain computed tomography did not reveal any abnormalities, although T2- and fluid-attenuated inversion recovery magnetic resonance imaging (MRI) revealed symmetrical hyperintensities in the bilateral parietal-occipital lobes (Fig. 1). Epileptiform discharge was not observed on electroencephalography. Based on these findings, RPLS and community-acquired pneumonia were highly suspected.

After the diagnosis of RPLS, thorough investigations were completed to identify possible triggers. The patient had none of the well-established RPLS risk factors, such as uncontrolled BP or immunosuppressant use. The BP was normal at both admission and sequencing monitoring, although BP before admission had not been recorded. Her symptoms improved after the administration of antiepileptic drugs and the antibiotic ceftriaxone for 5 days. These improvements were confirmed using laboratory investigations and chest radiography. The patient regained consciousness, and no new seizures were reported prior to discharge. A follow-up brain MRI after 3 months showed resolution of the previous abnormalities. A definitive diagnosis of RPLS was established. The patient did not experience seizures or adverse effects of drugs in a 2-year follow-up period. She provided informed consent for publication of the case.

## Discussion and conclusions

The present case demonstrates that RPLS can be triggered by infection in adults with nephrotic syndrome rather than by acute hypertension or immunosuppression. The patient was cured of her RPLS following immediate and appropriate antibiotic and antiepileptic treatment.

Although the present patient had chronic hypertension, her BP was stable without any abrupt elevations correlated with the onset of RPLS. Of note, although RPLS can be associated with kidney disease in various scenarios, particularly increased BP and calcineurin inhibitor use [4], this was not observed in the present patient. In fact, pneumonia development was the only variable that immediately preceded the onset of seizures.

The causes of RPLS vary, but its clinical symptoms are relatively common and can be attributed to several acute and chronic medical conditions, such as hypertension, immunosuppressive therapy, eclampsia, porphyria, vasculitis, and renal disease. However, nephrotic syndrome and infection, which were two causative factors in the present case, are rarely reported in the development of RPLS [1, 3]. Interestingly, nephrotic syndrome and infection-related RPLS may have similar pathogenesis related to endothelial dysfunction, which is typical of both hypertension and immunosuppressant-related RPLS.

Systemic BP elevation overwhelms the autoregulatory capacities of the brain vasculature, resulting in blood-brain barrier breakdown and allowing fluid and blood products into the brain parenchyma. This is known as vasogenic edema, and it is the usual proposed pathogenesis of RPLS [3]. Because there is sparse sympathetic innervation of the vertebrobasilar vascular system, the posterior regions of the brain may be more prone to failed cerebral autoregulation. Patients with nephrotic syndrome present with endothelial damage in the early stages of the disease, along with high levels of endothelial activation biomarkers, possibly induced by hypoalbuminemia or dyslipoproteinemia [5]. The cause of infection-related endothelial damage is both structural and functional [6]. Both infection and nephrotic syndrome affect endothelial function, subsequently impairing cerebral autoregulation and finally causing brain capillary leakage [3, 5]. Furthermore, these etiologies may appear in combination to cause more RPLS attacks, which is possibly why RPLS can be provoked by infection, as in our case of nephrotic syndrome without hypertension or immunosuppression.

Although the above models explain why vasogenic edema presents in images of most patients with RPLS, a small number also display cytotoxic edema, which is why most clinical symptoms of RPLS are reversible, although permanent neurological deficits or even death is occasionally observed [7]. The present study suggests that additional information should be gathered regarding RPLS in adults, and further studies on the potentially overlapping mechanisms of the different RPLS triggers should be conducted. Timely identification of RPLS, active control of BP and other inducing factors, and administration of antiepileptic drugs produce good prognosis.

RPLS can be provoked by infection in adults with nephrotic syndrome, as observed in our case. When such patients contract an infection, RPLS symptoms should be carefully monitored as early diagnosis and appropriate treatment can prevent morbidity and mortality.

## Data Availability

Not applicable.

## Abbreviations

RPLS: Reversible posterior leukoencephalopathy syndrome
MRI: Magnetic resonance imaging
BP: Blood pressure
